# Editorial: Cutaneous manifestations of systemic disease

**DOI:** 10.3389/fmed.2023.1236570

**Published:** 2023-06-20

**Authors:** Alvise Sernicola, Mauro Alaibac

**Affiliations:** Unit of Dermatology, Department of Medicine, University of Padua, Padua, Italy

**Keywords:** cutaneous, systemic disease, paraneoplastic pemphigus, tuberous sclerosis complex, pyoderma gangrenosum, calcinosis, gluten-related disorder, atypical exanthems

Clinical dermatology is commonly regarded as an “external medicine”. However, skin-directed approaches open a privileged window on the processes of internal diseases due to the established associations between cutaneous manifestations and systemic disease.

In oncology, paraneoplastic dermatoses may be the first sign of a previously unnoticed malignancy or an early warning of tumor recurrence after treatment. In their interesting study, Du et al. highlight the systemic nature of paraneoplastic pemphigus that may be viewed as a “paraneoplastic autoimmune multi-organ syndrome”. Paraneoplastic pemphigus is an uncommon life-threatening mucocutaneous syndrome that is frequently associated with non-Hodgkin lymphoma and chronic lymphocytic leukemia but not notably related to common solid organ tumors. Severe stomatitis refractory to treatment is the defining clinical feature, and the most unfavorable complication of this disease is respiratory involvement with bronchiolitis obliterans, which may prove fatal. The authors provide evidence supporting possible myocardial involvement associated with paraneoplastic pemphigus and hypothesize a shared immunopathogenesis. Paraneoplastic pemphigus may represent a challenging diagnosis, requiring clinical and laboratory testing together with evidence of an underlying neoplasm, and additional insight on its molecular pathogenesis will be required to increase our understanding of potential organ involvements beyond the skin.

In patients with genetic neurocutaneous disorders, skin changes—especially pigmentary lesions—appear very early, in many cases enabling dermatologists to raise clinical suspicion for diagnosis. In the case of tuberous sclerosis complex, a neurocutaneous syndrome with autosomal dominant inheritance, hypomelanotic macules are the first cutaneous finding in newborns and infants, while facial angiofibromas develop between the age of 2 years and adolescence. Hamartomas can develop in a variety of internal organs, requiring routine investigations in these patients to detect and treat potential complications. However, the management of facial angiofibromas cannot be disregarded as these visible lesions are associated with impaired quality of life and potential stigmatization. Monaghan et al. present the results of a survey study involving 235 patients living with tuberous sclerosis complex or their caregivers. The authors highlight a broad negative impact of facial angiofibromas on the quality of life and psychological wellbeing that also extends to the patients' caregivers. Moreover, the use of topical mammalian target of rapamycin inhibitors (rapamycin/sirolimus) was subjectively reported to be useful by two-thirds of the interviewed subjects, supporting the clinical benefit of this approach, which requires maintenance applications to prevent relapse. Additional physical approaches, such as laser ablation, are employed with variable results.

In rheumatologic and autoimmune disorders, disease-related skin lesions constitute red flags that may occur in association with a broad spectrum of systemic conditions. Pyoderma gangrenosum is a neutrophilic ulcerative disorder that occurs in patients presenting an associated disease in at least 50% of cases. Therefore, early identification and treatment of an underlying condition are key to the management of pyoderma gangrenosum, as is the correct clinical diagnosis, considering that laboratory and histologic features are non-specific and that other causes of skin ulceration must be ruled out. Chen et al. review the state of the art in diagnostics and therapeutics of pyoderma gangrenosum in light of their personal clinical experience, with a focus on local wound treatment and surgical approaches that maintain their role even in the current era of biologics and targeted agents. In this regard, monoclonal antibodies are known culprits of paradoxical skin reactions, including the occurrence or worsening of pyoderma gangrenosum. Romagnuolo et al. report on a case of pyoderma gangrenosum following the administration of tumor necrosis factor inhibitor adalimumab in a patient with chronic recurrent multifocal osteomyelitis. This contribution successfully highlights that associated risk factors may have additive effects in triggering the disease: clinicians are therefore reminded that certain medications can be responsible for drug-induced—or drug-exacerbated—disease and that numerous inheritable autoinflammatory disorders present pyoderma gangrenosum among their defining cutaneous features. Calcinosis, a deposition of insoluble calcium salts within cells of the skin and underlying tissues, is a dermatological complication that afflicts up to 40% of pediatric patients with juvenile dermatomyositis. Pinotti et al. effectively review current evidence on the pathogenesis and treatment of calcinosis, drawing attention to its major impact on how patients perceive the disease. Moreover, while randomized controlled trials have been performed in adults with dermatomyositis, their results are not automatically applicable to pediatric patients with this rare disorder, and treatment guidelines supported by rigorous studies are still lacking.

In addition, certain skin manifestations are considered disease-specific due to their invariable association with an established condition. Such has been the case of dermatitis herpetiformis with celiac disease, but this notion is recently expanding to include a broad skin spectrum of celiac disease and beyond. Verdelli et al. comprehensively review the cutaneous manifestations of gluten-related disorders, IgE-mediated wheat allergy, and non-celiac gluten sensitivity. Diagnostic and therapeutic guidelines are available for dermatitis herpetiformis but are lacking for other skin disorders related to gluten; therefore, constant cooperation between gastroenterologists and dermatologists is required for their management, which, in most cases, can benefit from a gluten-free diet. In the literature, skin manifestations have been reported in almost 70% of patients with wheat allergy, with their primary management relying on allergen avoidance, both food and inhaled, with additional strategies being desensitization with allergen-specific immunotherapy. Emergency medications are required for those at risk of anaphylaxis. Non-celiac gluten sensitivity defines a complex entity related to the intake of gluten that improves following a gluten-free diet and is the topic of a growing body of research. Cutaneous manifestations associated with non-celiac gluten sensitivity are reported in the literature as is their response to a gluten-free diet. Further evidence is needed to confirm these preliminary findings and novel pathogenic insights might be provided by investigating the skin and gut microbiota in these patients.

Finally, cutaneous tropism is a characteristic of several viruses, primarily human herpesviruses and lately SARS-CoV-2, but our understanding of the related skin manifestations is still incomplete. Michelerio et al. report on nine patients with demonstrated HHV-7 replication who presented with atypical exanthems. This topic is of broad interest considering that virus-related skin eruptions are often a reason for consultation in primary and urgent care settings. Atypical exanthems display clinical features that set them apart from the classic viral exanthems and, according to the authors' results, manifest with a maculopapular eruption that in some cases may be limited only to the acral sites.

## Author contributions

Conceptualization, writing—original draft, and writing—review and editing: AS and MA. All authors have read and agreed to the published version of the manuscript.

